# Functional Expression of IP, 5-HT_4_, D_1_, A_2A_, and VIP Receptors in Human Odontoblast Cell Line

**DOI:** 10.3390/biom13060879

**Published:** 2023-05-23

**Authors:** Eri Kitayama, Maki Kimura, Takehito Ouchi, Masahiro Furusawa, Yoshiyuki Shibukawa

**Affiliations:** 1 Department of Physiology, Tokyo Dental College, 2-9-18, Kanda-Misaki-cho, Chiyoda-ku, Tokyo 101-0061, Japan; kitayamaeri@tdc.ac.jp (E.K.);; 2 Department of Endodontics, Tokyo Dental College, 2-9-18, Kanda-Misaki-cho, Chiyoda-ku, Tokyo 101-0061, Japan

**Keywords:** odontoblasts, human, G_s_ protein-coupled receptor, adenylyl cyclase, immunofluorescence, cAMP dynamics

## Abstract

Odontoblasts are involved in sensory generation as sensory receptor cells and in dentin formation. We previously reported that an increase in intracellular cAMP levels by cannabinoid 1 receptor activation induces Ca^2+^ influx via transient receptor potential vanilloid subfamily member 1 channels in odontoblasts, indicating that intracellular cAMP/Ca^2+^ signal coupling is involved in dentinal pain generation and reactionary dentin formation. Here, intracellular cAMP dynamics in cultured human odontoblasts were investigated to understand the detailed expression patterns of the intracellular cAMP signaling pathway activated by the G_s_ protein-coupled receptor and to clarify its role in cellular functions. The presence of plasma membrane Gα_s_ as well as prostaglandin I_2_ (IP), 5-hydroxytryptamine 5-HT_4_ (5-HT_4_), dopamine D_1_ (D_1_), adenosine A_2A_ (A_2A_), and vasoactive intestinal polypeptide (VIP) receptor immunoreactivity was observed in human odontoblasts. In the presence of extracellular Ca^2+^, the application of agonists for the IP (beraprost), 5-HT_4_ (BIMU8), D_1_ (SKF83959), A_2A_ (PSB0777), and VIP (VIP) receptors increased intracellular cAMP levels. This increase in cAMP levels was inhibited by the application of the adenylyl cyclase (AC) inhibitor SQ22536 and each receptor antagonist, dose-dependently. These results suggested that odontoblasts express G_s_ protein-coupled IP, 5-HT_4_, D_1_, A_2A_, and VIP receptors. In addition, activation of these receptors increased intracellular cAMP levels by activating AC in odontoblasts.

## 1. Introduction

Plasma membrane ‘multistimulatory receptor proteins’, which are capable of receiving various external and internal stimuli, can be categorized as follows: G protein-coupled (metabotropic) receptors (GPCRs) or ligand-gated ion channels (ionotropic receptors). GPCRs activate the intracellular second messenger pathway to transmit cellular information via G protein activation. These G proteins are composed of α, β, and γ subunits and are identified by their Gα subunits. They are typically classified into four families; Gα_s_, Gα_i_, Gα_q_, and Gα_12/13_ [1,2], which are expressed in most cell types. There are two principal intracellular signaling pathways induced by GPCR activation: the cAMP and phosphatidylinositol signaling pathways. Both Gα_s_ and Gα_i_ affect cAMP-generating enzyme adenylyl cyclase (AC), whereas Gα_q_ activates phospholipase Cβ, which divides phosphatidylinositol 4,5-bisphosphate into diacylglycerol and inositol 1,4,5-trisphosphate. Ligand binding to G_s_ protein-coupled receptors activates AC to catalyze the conversion of ATP to cAMP. cAMP regulates the actions of the four following proteins: protein kinase A, cAMP-dependent exchange protein, cyclic nucleotide-gated channels, and Popeye domain-containing proteins, and these specific downstream cAMP effectors play important roles in the regulation of various physiological functions [3,4], such as the regulation of hormone synthesis, thyroid cell mitogenesis, bone resorption, and cardiac excitation/contraction coupling by the sympathetic nervous system [2].

Ca^2+^ influx induced by activation of transient receptor potential (TRP) channels, such as TRP vanilloid subfamily member 1 (TRPV1), and by activation of mechanosensitive Piezo1 channels in odontoblasts plays a critical role in producing and transmitting sensations to dentin (‘odontoblast mechanosensory/hydrodynamic receptor model’) as well as in developmental, physiological, and pathological dentin formation [5,6,7]. The application of various stimuli, including thermal, osmotic, and chemical stimuli, induces membrane deformation via dentinal fluid movement. Membrane deformation is detected as a mechanical stimulus via TRPV1, TRPV2, TRPV4, TRP ankyrin subfamily member-1 (TRPA1), and Piezo channels in odontoblasts [5,6,7,8]. Ca^2+^ influx via the TRP and Piezo1 channels acts as the primary intracellular signal for ATP release from pannexin-1 (PANX-1) and glutamate from glutamate-permeable anion channels. The released ATP and glutamate act as neurotransmitters and activate the P2X receptor subtype 3 and metabotropic glutamate receptors in pulpal neurons, respectively. The activation of these receptors is involved in generating dentinal pain [6,7,9,10]. Functional crosstalk between cannabinoid 1 (CB1) receptors and TRPV1 channels in odontoblasts has also been demonstrated. CB1 receptors functionally couple primarily with G_i_- and G_s_-mediated pathways to regulate intracellular cAMP levels [11]. The application of 2-arachidonyl glycerol (2-AG) (a nonselective CB1 and CB2 receptor agonist, but not a TRPV1 agonist) increases the intracellular free Ca^2+^ concentration ([Ca^2+^]_i_) in rat odontoblasts. The increase in 2-AG-induced [Ca^2+^]_i_ is significantly inhibited by a TRPV1 and TRP melastatin subfamily member 8 (TRPM8) channel antagonist, whereas a specific TRPM8 channel antagonist does not affect the increase in [Ca^2+^]_i_. In addition, this increase is significantly suppressed by a selective CB1 receptor antagonist and AC inhibitor, whereas no effect has been observed with a selective CB2 receptor antagonist. These results suggest that cAMP signals are produced by AC, which is activated by the CB1 receptor, resulting in enhanced Ca^2+^ influx via TRPV1 channel activation in odontoblasts [8]. These reports imply that intracellular cAMP levels are capable of mediating Ca^2+^ signaling and may participate in dentin formation and dentinal pain.

In addition, recent studies have shown that rat odontoblasts are immunoreactive to G_s_ protein-coupled β_2_ and calcitonin gene-related peptide (CGRP) receptor antibodies [12,13], and mouse odontoblasts express the parathyroid hormone receptor as observed in in situ hybridization assays [14]. The immunofluorescent expression of prostaglandin (PG) I_2_ (IP) receptors [15] and the mRNA expression of dopamine (DA) D_1_ (D_1_) receptors in rat odontoblasts have also been reported [16]. Human dental pulp cells express mRNAs of all four adenosine receptor subtypes (A_1_, A_2A_, A_2B_, and A_3_ receptors) in RT-PCR analysis. The expression levels of G_s_ protein-coupled A_2A_ and G_s/q_ protein-coupled A_2B_ receptor mRNA are higher than those of G_i/o_ protein-coupled A_1_ and A_3_ receptors [17]. Furthermore, the existence of nerve endings that are located near odontoblasts and that contain vasoactive intestinal polypeptide (VIP) implies the expression of VPAC_1_ and VPAC_2_ as subtypes of VIP receptors in odontoblasts [18]. Moreover, the 5-hydroxytryptamine (5-HT) receptors are involved in enamel morphogenesis and maturation in mice [19]. Among them, 5-HT_4_ is a ubiquitously expressed G_s_ protein-coupled receptor. However, the functional expression of G_s_ protein-coupled (IP, D_1_, A_2A_, VPAC_1/2_, and 5-HT_4_) receptors and the detailed intracellular cAMP signaling pathway following their receptor activation in odontoblasts remain unclear. 

In the present study, we selected specific G_s_ protein-coupled receptors for screening based on their known morphological and mRNA expression patterns in odontoblasts and their anatomical/functional relationships; the intracellular cAMP dynamics involving these receptors were assessed in single, living odontoblasts.

## 2. Materials and Methods

### 2.1. Cell Culture

A human odontoblast cell line was obtained from a healthy third molar and immortalized by transfection with the human telomerase transcriptase gene [20,21,22]. This cell line represents a pure population of cells with odontoblast properties and exhibits the mRNA expression of dentin sialophosphoprotein (DSPP), type 1 collagen, and alkaline phosphatase [20]. Human odontoblasts were cultured in basal medium (pH 7.4) (alpha-minimum essential medium containing 10% fetal bovine serum, 100 U/mL penicillin-streptomycin (Thermo Fisher Scientific Inc., Waltham, MA, USA), and amphotericin B (Sigma-Aldrich, St. Louis, MO, USA)) at 37 °C in a 5% CO_2_ incubator for 48 h. The odontoblast suspension was adjusted to a density of 5 × 10^4^ cells/mL.

### 2.2. Immunofluorescence

Human odontoblasts were cultured in eight-well glass chambers (Iwaki, Shizuoka, Japan) and maintained at 37 °C and 5% CO_2_. The cells were fixed with 4% paraformaldehyde (FUJIFILM Wako Pure Chemical Co., Osaka, Japan) and washed with 1× phosphate-buffered saline (PBS; Thermo Fisher Scientific, Inc.). Cells were permeabilized in PBS containing 0.1% Triton-X100 for 5 min. After 10–15 min of incubation with blocking buffer (Nacalai Tesque, Kyoto, Japan) at room temperature, the following primary antibodies were applied for 3–4 h, also at room temperature or overnight at 4 °C: rabbit polyclonal anti-DSPP (Bioss, Woburn, MA, USA; bs-8557R, 1:200), mouse monoclonal anti-nestin (Santa Cruz Biotechnology, Inc., Dallas, TX, USA; sc-23927, 10c2, 1:200), and mouse monoclonal anti-dentin matrix protein 1 (anti-DMP-1; Santa Cruz Biotechnology, Inc.; sc-73633, LFMb-31, 1:200), as odontoblast markers. Human odontoblasts were also stained immunohistochemically for the G_s_ protein-coupled receptors using the following primary antibodies: rabbit polyclonal anti-guanine nucleotide binding Gα_s_ (ABclonal, Tokyo, Japan; A5546, 1:200), mouse monoclonal anti-A_2A_ receptor (Santa Cruz Biotechnology, Inc., sc-32261, 7F6-G5-A2, 1:200), mouse monoclonal anti-D_1_DR (Santa Cruz Biotechnology, Inc., sc-33660, SG2-D1a, 1:200), mouse monoclonal anti-IP receptor (Santa Cruz Biotechnology, Inc., sc-365268, B-3, 1:200), mouse monoclonal anti-VPAC_1_ (Santa Cruz Biotechnology, Inc., sc-377152, B-4, 1:200), mouse monoclonal anti-VPAC_2_ (Santa Cruz Biotechnology, Inc., sc-52795, AS69, 1:200), and rabbit polyclonal anti-5-HT_4_ receptor (Bioss, bs-2127R, 1:200). Western blotting data is commercially available to validate the specificity of the primary antibodies for mouse monoclonal anti-A_2A_ receptor, rabbit polyclonal anti-5-HT_4_ receptor, mouse monoclonal anti-D_1_DR, rabbit polyclonal anti-guanine nucleotide binding Gα_s_, mouse monoclonal anti-IP receptor, mouse monoclonal anti-VPAC_1_, mouse monoclonal anti-nestin, and mouse monoclonal anti-DMP-1. Previous studies have also validated the specificity of mouse monoclonal anti-VPAC_2_ and rabbit polyclonal anti-DSPP via Western blotting [23,24,25]. The secondary antibodies were applied for 1 h at room temperature. The following secondary antibodies were used for immunostaining: Alexa Fluor^®^ 488 donkey anti-mouse (Thermo Fisher Scientific, Inc.), Alexa Fluor^®^ 488 donkey anti-rabbit, and Alexa Fluor^®^ 568 donkey anti-rabbit (Thermo Fisher Scientific, Inc.) antibodies. To verify the specificity of the immunoreactions, staining was controlled by omitting primary antibodies from the first incubation fluid. The stained samples were mounted in a mounting medium containing 4,6-diamidino-2-phenylindole (Abcam, Cambridge, UK). Images of immunostaining were obtained using a fluorescence microscope (BZ-X710; Keyence, Osaka, Japan).

### 2.3. Solutions and Reagents

A standard solution containing 136 mM NaCl, 5 mM KCl, 2.5 mM CaCl_2_, 0.5 mM MgCl_2_, 10 mM HEPES, 10 mM glucose, and 12 mM NaHCO_3_ (pH 7.4) was used as the extracellular solution for intracellular cAMP level measurements. Beraprost sodium, Ro1138452 hydrochloride, BIMU8, GR113808, SKF83959 hydrobromide, LE300, PSB0777 ammonium salt, ZM241385, VIP (human, rat, mouse, rabbit, canine, and porcine), and VIP(6-28) (human, rat, porcine, and bovine) were obtained from TOCRIS Bioscience (Bristol, UK). Stock solutions were prepared in dimethyl sulfoxide for beraprost, Ro1138452, BIMU8, GR113808, SKF83959, LE300, PSB0777, and ZM241385, and in MilliQ water for VIP and VIP(6-28). Stock solutions were diluted with standard solutions to the appropriate concentrations before use.

### 2.4. Measurement of Intracellular cAMP Level

For the live-cell cAMP sensor assay, the green upward cAMP BacMam sensor (green upward cAMP difference detector in situ; Montana Molecular, Bozeman, MT, USA) was used. The green upward cAMP BacMam sensor is a vector supplied as a nonreplicating baculovirus expressed by infecting the cells. The vector contains a gene encoding a fluorescent protein that functions as a transient cAMP-sensitive fluorescent biosensor. The fluorescence intensity of the protein increases when the vector specifically binds to cAMP in live mammalian cells. The cAMP sensor was transfected in human odontoblasts by incubation in basal medium containing 16.7% BacMam sensor and 0.4% Na-butyrate at 37 °C for 36 h. BacMam-sensor-transfected human odontoblasts were rinsed with fresh standard solution and then mounted on a dish on the stage of a microscope (IX73, Olympus, Tokyo, Japan) incorporated with HCImage software, an excited wavelength selector, and an intensified charge-coupled device camera system (Hamamatsu Photonics, Shizuoka, Japan). The green fluorescence emission was measured at 517 nm at an excitation wavelength of 506 nm. The intracellular cAMP levels were expressed as the fluorescence intensity ratio (F/F_0_ unit) of the fluorescence intensity (F) to the resting value (F_0_). The F/F_0_ baseline (F/F_0 baseline_) was denoted as the mean value for 30 s before the first application of each receptor agonist and was set at 1.0. To evaluate the pharmacological effect of the G_s_ protein-coupled receptor antagonist on its agonist-induced increase in cAMP levels, we first calculated the ΔF value by the following equation,

ΔF = (F/F_0 peak_) − (F/F_0 baseline_)

where F/F_0 peak_ is a peak F/F_0_ value and F/F_0 baseline_ is F/F_0_ baseline by G_s_ protein-coupled receptor agonist application with or without its antagonist. We then normalized the ΔF value during agonist application with (ΔF_antagonist_) or without (ΔF_agonist_ as 100%) antagonist application. A standard solution with or without each receptor agonist, antagonist, or SQ22536 was applied via superfusion using a rapid gravity-fed perfusion system (ValueLink8.2 Controller; AutoMate Scientific, Barkeley, CA, USA). A series of repeated applications (2 min) of each receptor agonist with or without the antagonist or SQ22536 was applied to the cells, and they were washed with standard solution until the F/F_0_ value returned to baseline. All experiments were performed at 28 ± 1 °C.

### 2.5. Statistical Analysis

Data are expressed as the mean ± standard error of the mean of N observations, where N represents the number of independent experiments. One-way ANOVA with Tukey’s post-hoc test was used to determine the parametric statistical significance. Statistical significance was set at *p* < 0.05. All statistical analyses were performed using GraphPad Prism 7.0 software (GraphPad Software, La Jolla, CA, USA).

## 3. Results

### 3.1. Immunofluorescence Analysis of Human Odontoblast Markers

**Figure 1:**
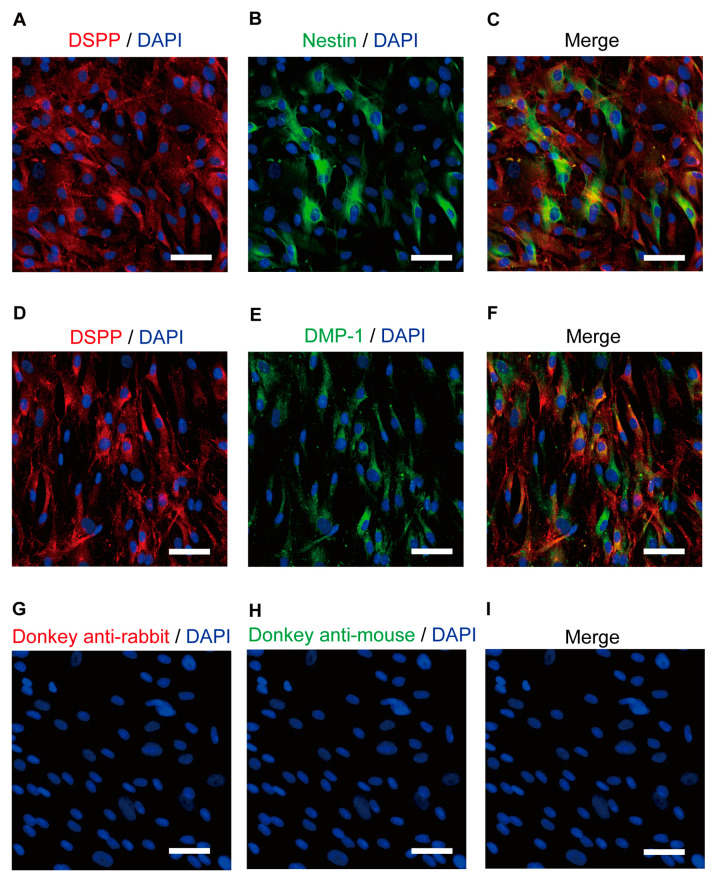
Immunofluorescence analysis of the DSPP, nestin, and DMP-1 human odontoblast markers. (**A**,**B**,**D**,**E**) Human odontoblasts showed positive immunoreactivity to the dentin sialophosphoprotein (DSPP) (red in (**A**,**D**)), nestin (green in (**B**)), and dentin matrix protein 1 (DMP-1) (green in (**E**)). (**C**,**F**) Triple immunofluorescence staining with antibodies against the DSPP (red) and nestin (green) (**C**) or DMP-1 (green) (**F**). (**G**–**I**) No fluorescence was detected in the negative control omitting the primary antibody to control the specificity of the immunostaining with Alexa 488 and/or 568. Nuclei are shown in blue. Scale bars; 50 µm.

Immunoreactivity for DSPP (Figure 1A,C,D,F), the intermediate filament protein nestin (Figure 1B,C), and DMP-1 (Figure 1E,F), which are marker proteins in human odontoblasts, were observed, indicating that the cells used were odontoblasts.

### 3.2. Human Odontoblasts Were Immunopositive for Gα_s_ Protein and IP, 5-HT_4_, D_1_, A_2A_, and VIP Receptor Antibodies

**Figure 2:**
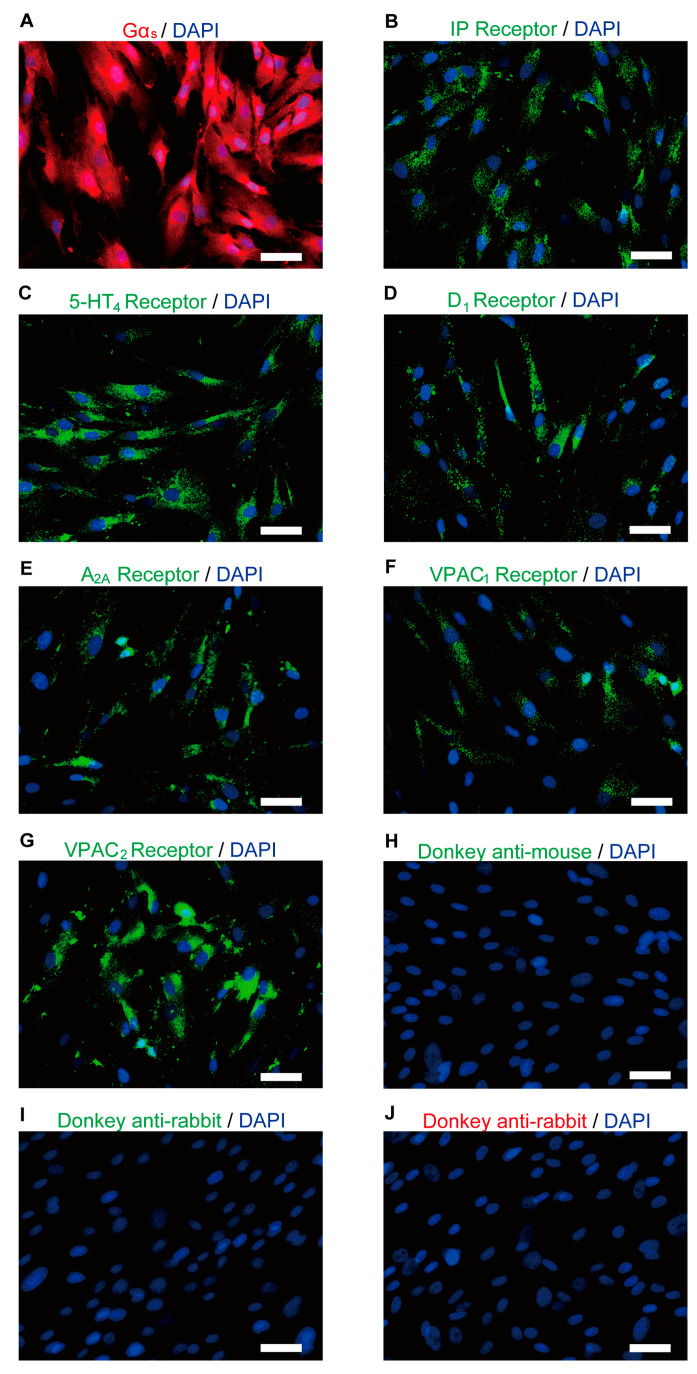
Immunofluorescence analysis of Gα_s_, IP, 5-HT_4_, D_1_, A_2A_, and VIP receptors in human odontoblasts. (**A**–**G**) Human odontoblasts showed positive immunoreactivity to Gα_s_ (red in (**A**)), IP (green in (**B**)), 5-HT_4_ (green in (**C**)), D_1_ (green in (**D**)), A_2A_ (green in (**E**)), VPAC_1_ (green in (**F**)), and VPAC_2_ receptors (green in (**G**)). (**H**–**J**) No fluorescence was detected in the negative control omitting the primary antibody to control the specificity of the immunostaining with Alexa 488 or 568. Nuclei are shown in blue. Scale bars; 50 µm.

Immunofluorescence analysis revealed that the human odontoblasts were immunopositive for Gα_s_ protein (Figure 2A) and for IP (Figure 2B), 5-HT_4_ (Figure 2C), D_1_ (Figure 2D), and A_2A_ (Figure 2E) receptors. The odontoblasts also showed immunoreactivity for VPAC_1_ (Figure 2F) and VPAC_2_ (Figure 2G), which are subtypes of the VIP receptor. 

### 3.3. The IP Receptor Agonist Increased Intracellular cAMP Levels in Odontoblasts

**Figure 3:**
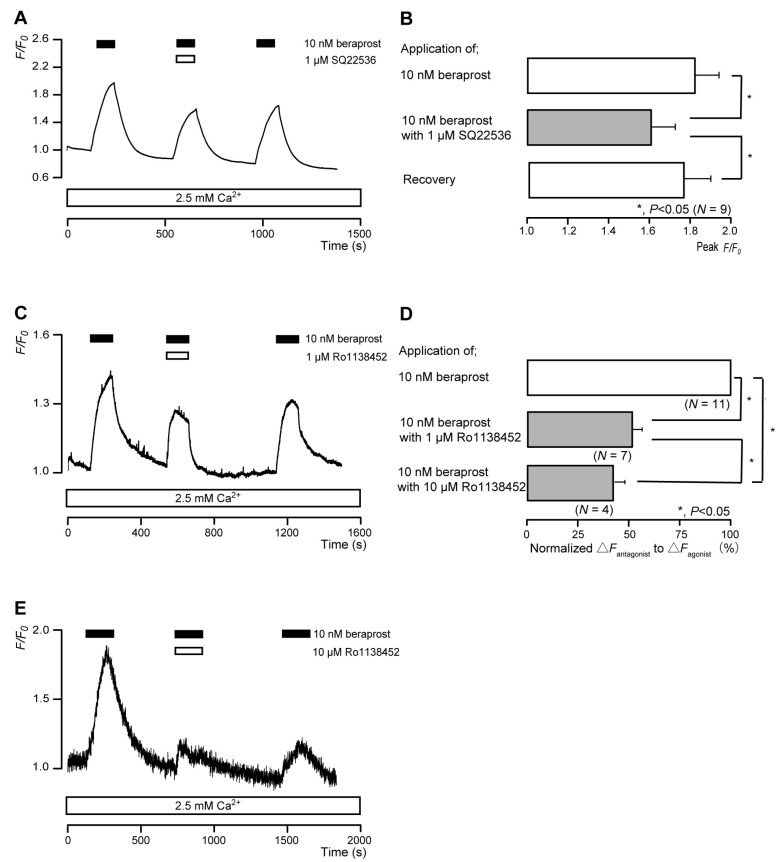
IP receptor-agonist-induced intracellular cAMP level increases. (**A**,**C**,**E**) Representative traces of transient intracellular cAMP level increases in response to 10 nM beraprost, with or without 1 µM SQ22536 (**A**), 1 µM Ro1138452 (**C**), or 10 µM Ro1138452 (**E**), in the presence of extracellular Ca^2+^ (2.5 mM) (white boxes at bottom). Black boxes at the top indicate periods of beraprost addition to the external solution. White boxes at the top indicate periods of SQ22536 (**A**) or Ro1138452 (**C**,**E**) addition to the external solution. (**B**) Bar graphs of beraprost-induced intracellular cAMP level increases, without (**upper** column) or with (**middle** column) 1 µM SQ22536. Recovery (**lower** column) shows reversible effect of SQ22536. (**D**) Bar graphs of inhibitory effects of 1 µM Ro1138452 (**middle** column) or 10 µM Ro1138452 (**lower** column), having normalized 10 nM beraprost-induced increases in the intracellular cAMP in the presence of Ro1138452 to that in the absence of Ro1138452. Each bar in (**B**,**D**) denotes the mean ± standard error (SE) values across the number of experiments shown in parenthesis. Statistically significant differences between columns (solid lines) are indicated with asterisks: * *p* < 0.05.

The intracellular cAMP levels in human odontoblasts were measured using an mNeon Green-based cAMP sensor. The application of 10 nM beraprost (a potent IP receptor agonist) to human odontoblasts transiently increased intracellular cAMP levels, which reached peak values of 1.82 ± 0.11 F/F_0_ units (*N* = 9; Figure 3A,B), 1.43 ± 0.03 F/F_0_ units (*N* = 7; Figure 3C), or 1.30 ± 0.16 F/F_0_ units (*N* = 4; Figure 3E). Beraprost-induced intracellular cAMP level increases were significantly and reversibly inhibited by an AC inhibitor (1 µM SQ22536). In addition, these heightened cAMP levels were significantly suppressed by a selective IP receptor antagonist (Ro1138452) to 51.8 ± 4.39% (*N* = 7; Figure 3C,D) with a 1 μM application and to 42.0 ± 5.55% (*N* = 4; Figure 3D,E) with a 10 μM application. Intracellular cAMP levels were recovered by removing SQ22536 and the IP receptor antagonist.

### 3.4. The 5-HT_4_ Receptor Agonist Increased Intracellular cAMP Levels in Odontoblasts

**Figure 4:**
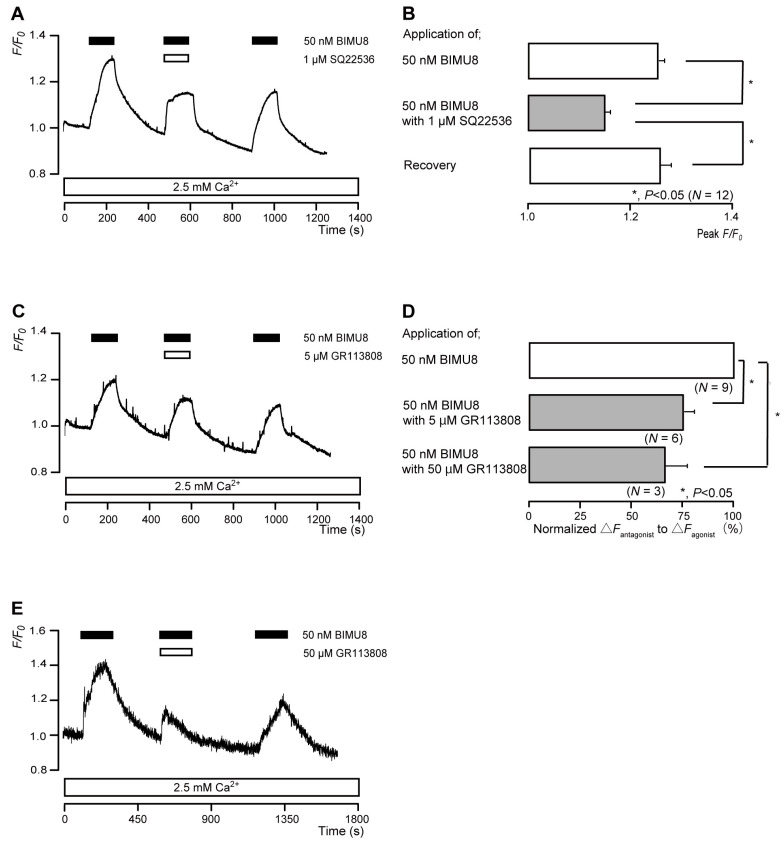
5-HT_4_ receptor-agonist-induced intracellular cAMP level increases. (**A**,**C**,**E**) Representative traces of transient intracellular cAMP level increases in response to 50 nM BIMU8, with or without 1 µM SQ22536 (**A**), 5 µM GR113808 (**C**), or 50 µM GR113808 (**E**), in the presence of extracellular Ca^2+^ (2.5 mM) (white boxes at bottom). Black boxes at the top indicate periods of BIMU8 addition to the external solution. White boxes at the top indicate periods of SQ22536 (**A**) or GR113808 (**C**,**E**) addition to the external solution. (**B**) Bar graphs of BIMU8-induced intracellular cAMP level increases, without (**upper** column) or with (**middle** column) 1 µM SQ22536. Recovery (**lower** column) shows reversible effect of SQ22536. (**D**) Bar graphs of inhibitory effects of 5 µM GR113808 (**middle** column) or 50 µM GR113808 (**lower** column) on the normalized value of 50 nM BIMU8-induced increases in the intracellular cAMP in the presence of GR113808 to that in the absence of GR113808. Each bar in (**B**,**D**) denotes the mean ± SE across the number of experiments shown in parenthesis. Statistically significant differences between columns (solid lines) are indicated with asterisks: * *p* < 0.05.

The application of 50 nM BIMU8 (a potent 5-HT_4_ receptor agonist) to human odontoblasts transiently increased intracellular cAMP levels to peak values of 1.26 ± 0.01 F/F_0_ units (*N* = 12; Figure 4A,B), 1.18 ± 0.02 F/F_0_ units (*N* = 6; Figure 4C), or 1.43 ± 0.18 F/F_0_ units (*N* = 3; Figure 4E). BIMU8-induced intracellular cAMP level increases were significantly and reversibly inhibited by an AC inhibitor (1 µM SQ22536). In addition, the application of selective receptor antagonist (GR113808) significantly suppressed BIMU8-induced increases in intracellular cAMP level to 74.3 ± 6.29% (*N* = 6; Figure 4C,D) with a 5 μM application and to 65.8 ± 10.8% (*N* = 3; Figure 4D,E) with a 50 μM application. Intracellular cAMP levels were recovered by removing SQ22536 and the 5-HT_4_ receptor antagonist.

### 3.5. The D_1_ Receptor Agonist Increased Intracellular cAMP Levels in Odontoblasts

**Figure 5:**
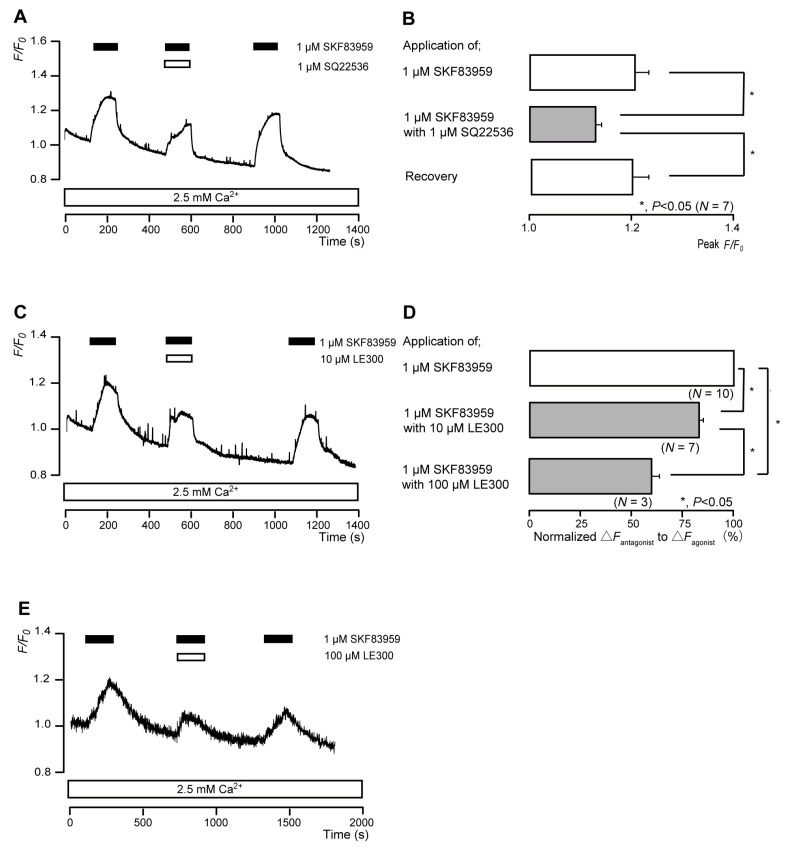
D_1_ receptor-agonist-induced intracellular cAMP level increases. (**A**,**C**,**E**) Representative traces of transient intracellular cAMP level increases in response to 1 μM SKF83959, with or without 1 µM SQ22536 (**A**), 10 µM LE300 (**C**), or 100 µM LE300 (**E**), in the presence of extracellular Ca^2+^ (2.5 mM) (white boxes at bottom). Black boxes at the top indicate periods of SKF83959 addition to the external solution. White boxes at the top indicate periods of SQ22536 (**A**) or LE300 (**C**,**E**) addition to the external solution. (**B**) Bar graphs of SKF83959-induced intracellular cAMP level increases, without (**upper** column) or with (**middle** column) 1 µM SQ22536. Recovery (**lower** column) shows the reversible effect of SQ22536. (**D**) Bar graphs of inhibitory effects of 10 µM LE300 (**middle** column) or 100 µM LE300 (**lower** column) on the normalized value of 10 μM SKF83959-induced increases in the intracellular cAMP in the presence of LE300 to that in the absence of LE300. Each bar in (**B**,**D**) denotes the mean ± SE across the number of experiments shown in parenthesis. Statistically significant differences between columns (solid lines) are indicated with asterisks: * *p* < 0.05.

The application of 1 μM SKF83959 (a partial D_1_ receptor agonist) to human odontoblasts transiently increased intracellular cAMP levels to peak values of 1.21 ± 0.03 F/F_0_ units (*N* = 7; Figure 5A,B), 1.22 ± 0.22 F/F_0_ units (*N* = 7; Figure 5C), or 1.45 ± 0.22 F/F_0_ units (*N* = 3; Figure 5E). SKF83959-induced intracellular cAMP level increases were significantly and reversibly inhibited by an AC inhibitor (1 µM SQ22536). In addition, a potent D_1_ receptor antagonist (LE300) significantly suppressed SKF83959-induced increases in intracellular cAMP level to 83.0 ± 2.38% (*N* = 7; Figure 5C,D) with a 10 μM application and to 59.4 ± 3.59% (*N* = 3; Figure 5D,E) with a 100 μM application. Intracellular cAMP levels were recovered by removing SQ22536 and the D_1_ receptor antagonist.

### 3.6. The A_2A_ Receptor Agonist Increased Intracellular cAMP Levels in Odontoblasts

**Figure 6:**
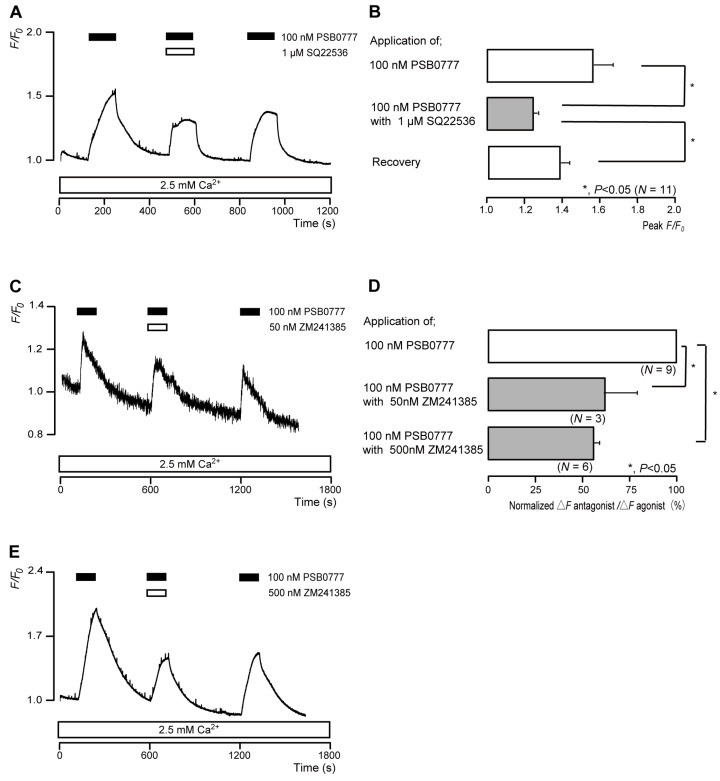
A_2A_ receptor-agonist-induced intracellular cAMP level increases. (**A**,**C**,**E**) Representative traces of transient intracellular cAMP level increases in response to 100 nM PSB0777, with or without 1 µM SQ22536 (**A**), 50 nM ZM241385 (**C**), or 500 nM ZM241385 (**E**), in the presence of extracellular Ca^2+^ (2.5 mM) (white boxes at bottom). Black boxes at the top indicate periods of PSB0777 addition to the external solution. White boxes at the top indicate periods of SQ22536 (**A**) or ZM241385 (**C**,**E**) addition to the external solution. (**B**) Bar graphs of PSB0777-induced intracellular cAMP level increases, without (**upper** column) or with (**middle** column) 1 µM SQ22536. Recovery (**lower** column) shows the reversible effect of SQ22536. (**D**) Bar graphs of inhibitory effects of 50 nM ZM241385 (**middle** column) or 500 nM ZM241385 (**lower** column) on the normalized value of 100 nM PSB0777-induced increases in the intracellular cAMP in the presence of ZM241385 to that in the absence of ZM241385. Each bar in (**B**,**D**) denotes the mean ± SE across the number of experiments shown in parenthesis. Statistically significant differences between columns (solid lines) are indicated with asterisks: * *p* < 0.05.

The application of 100 nM PSB0777 (a potent A_2A_ receptor agonist) to human odontoblasts transiently increased intracellular cAMP levels to peak values of 1.57 ± 0.10 F/F_0_ units (*N* = 11; Figure 6A,B), 1.29 ± 0.02 F/F_0_ units (*N* = 3; Figure 6C), or 1.99 ± 0.09 F/F_0_ units (*N* = 6; Figure 6E). PSB0777-induced intracellular cAMP level increases were significantly and reversibly inhibited by an AC inhibitor (1 µM SQ22536). In addition, a potent and selective A_2A_ receptor antagonist (ZM241385) significantly suppressed PSB0777-induced increases in intracellular cAMP level to 61.0 ± 16.9% (*N* = 3; Figure 6C,D) with a 50 nM application and to 54.6 ± 3.93% (*N* = 6; Figure 6D,E) with a 500 nM application. Intracellular cAMP levels were recovered by removing SQ22536 and the A_2A_ receptor antagonist.

### 3.7. The Nonselective VIP Receptor Agonist Increased Intracellular cAMP Levels in Odontoblasts

**Figure 7:**
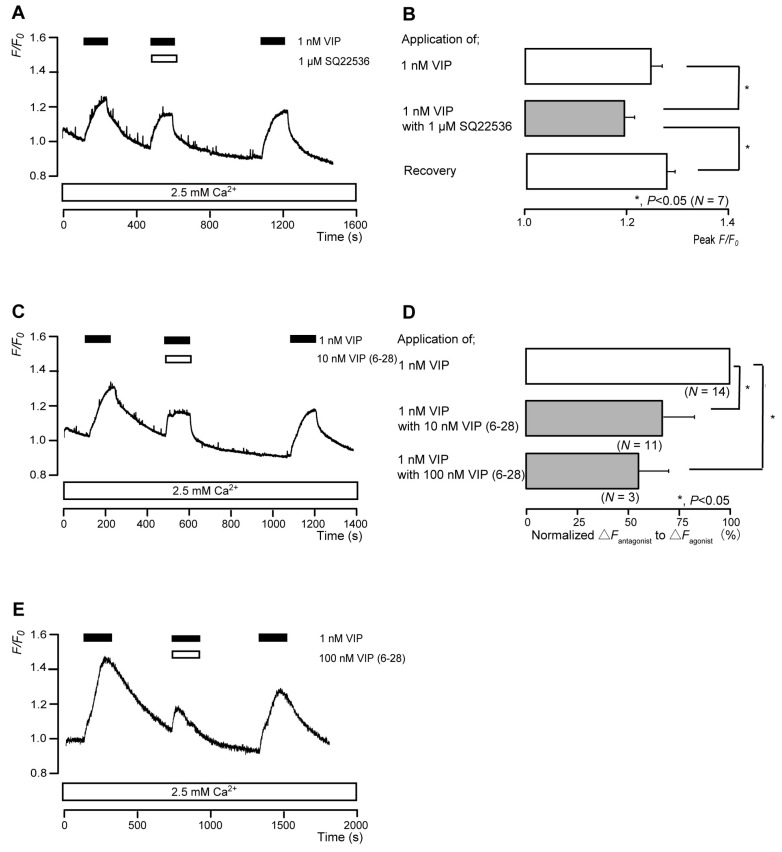
VIP receptor-agonist-induced intracellular cAMP level increases. (**A**,**C**,**E**) Representative traces of transient intracellular cAMP level increases in response to 1 nM VIP, with or without 1 µM SQ22536 (**A**), 10 nM VIP(6-28) (**C**), or 100 nM VIP(6-28) (**E**), in the presence of extracellular Ca^2+^ (2.5 mM) (white boxes at bottom). Black boxes at the top indicate periods of VIP addition to the external solution. White boxes at the top indicate periods of SQ22536 (**A**) or VIP(6-28) (**C**,**E**) addition to the external solution. (**B**) Bar graphs of VIP-induced intracellular cAMP level increases, without (**upper** column) or with (**middle** column) 1 µM SQ22536. Recovery (**lower** column) shows the reversible effect of SQ22536. (**D**) Bar graphs of inhibitory effects of 10 nM VIP(6-28) (**middle** column) or 100 nM VIP(6-28) (**lower** column) on the normalized value of 1 nM VIP-induced increases in the intracellular cAMP in the presence of VIP(6-28) to that in the absence of VIP(6-28). Each bar in (**B**,**D**) denotes the mean ± SE across the number of experiments shown in parenthesis. Statistically significant differences between columns (shown by solid lines) are indicated with asterisks: * *p* < 0.05.

The application of 1 nM VIP transiently increased intracellular cAMP levels to peak values of 1.25 ± 0.02 F/F_0_ units (*N* = 7; Figure 7A,B), 1.30 ± 0.01 F/F_0_ units (*N* = 11; Figure 7C), or 1.45 ± 0.06 F/F_0_ units (*N* = 3; Figure 7E). VIP-induced intracellular cAMP level increases were significantly and reversibly inhibited by an AC inhibitor (1 µM SQ22536). In addition, a nonselective VIP receptor antagonist (VIP(6-28)) significantly suppressed VIP-induced increases in intracellular cAMP level to 66.4 ± 5.48% (*N* = 11; Figure 7C,D) with a 10 nM application and to 55.8 ± 14.2% (*N* = 3; Figure 7D,E) with a 100 nM application. Intracellular cAMP levels were recovered by removing SQ22536 and the VIP receptor antagonist.

## 4. Discussion

Here, the functional expression of the Gα_s_ protein and the G_s_ protein-coupled IP, 5-HT_4_, D_1_, A_2A_, and VIP receptors in human odontoblasts was demonstrated. The activation of these Gα_s_ protein-coupled receptors regulates the AC signal transduction pathway to induce cAMP production.

Prostanoids are cyclooxygenase metabolites of arachidonic acid and include PGs, PGD_2_, PGE_2_, PGF_2α_, PGI_2_, and thromboxane A_2_. These are synthesized and released upon cell stimulation and act on cells in the vicinity of their synthesis to exert their actions [26]. They also belong to a subclass of eicosanoids consisting of the prostaglandins, thromboxanes, and prostacyclins. Prostaglandins have a broad range of pathophysiological effects, including a contribution to inflammation and pain perception. The specific receptors for PGD_2_, PGE_2_, PGF_2_, PGI_2_, and TXA_2_ are termed the DP, EP, FP, IP, and TP receptors, respectively [26]. In human embryonic kidney 293 (HEK293) cells expressing IP receptors, beraprost dose-dependently increased cAMP levels with a half-maximal (50%) effective concentration (EC_50_) of 10.4 nM [27]. SQ22536 dose-dependently blocked cAMP level increase during platelet aggregation with a half-maximal (50%) inhibitory concentration (IC_50_) of 1 µM [28]. SQ22536 (1 µM) also suppressed the 2-AG-induced increase in [Ca^2+^]_i_ in rat odontoblasts [8]. Ro1138452 is a potent and selective antagonist that has a high affinity for human IP receptors, but showed no significant potency for non-IP prostanoid receptors (EP_1_, EP_3_, FP, and TP receptors) [29]. The IC_50_ of Ro1138452 was 100 nM in IP-receptor-expressing CHO-K1 cells [29]. However, Ro1138452 has been used at higher concentrations, such as 1–10 µM, than the IC_50_ [30,31]. In this study, 10 nM beraprost, 1 µM SQ22536, and 1 µM Ro1138452 were used. Each concentration of these reagents was appropriate for activating or inhibiting the IP receptors or AC. PGI_2_ synthase (PGIS) immunoreactivity has been detected in odontoblasts and has been reported to increase with mechanical stimulation and maturation [32]. An experimental force was previously applied to the maxillary first and second molars by inserting an elastic band between them for 6–72 h; thereafter, the mRNA levels of PGIS, IP receptor, and TRPV1 were shown to be upregulated in rat molar pulp. In addition, rat odontoblasts showed PGIS, IP receptor, and TRPV1 immunoreactivities in that study, indicating the contribution of the PGIS–IP-receptor–TRPV1 axis to functional cellular processes in odontoblasts [15]. Extracellular PGI_2_ also potentiates/sensitizes TRPV1 channel activity via IP receptor activation and cAMP-dependent protein kinase A activity [33]. In our previous research, we found that TRPV1 channel activity (which is sensitive to mechanical stimulation) is closely involved in the generation of dentinal pain. Additionally, cAMP signals enhance Ca^2+^ influx via TRPV1 channel activation in odontoblasts [8]. Although further studies are required in this regard, IP receptor activation by extracellular PGI_2_ during inflammatory responses in dental pulp may modulate dentinal pain intensity by modulating mechanosensitive ion channels via cAMP signaling. Furthermore, Iloprost—a stable, long-acting PGI_2_ analog—enhances the mineralization of human dental pulp cells and reactionary dentin formation [34]. Our ongoing experiments aim to clarify the contribution of the IP receptor to odontoblast cellular functions, including dentin formation and sensory transduction in dentinal sensitivity.

DA, which is synthesized by tyrosine hydroxylase (TH), is the predominant catecholamine neurotransmitter in the brain, and it affects many physiological functions, such as the control of coordinated movements and hormone secretion as well as motivational and emotional behaviors. DA binds to DA receptors (D_1–5_ receptors), which are classified as either D_1_-like receptors (D_1_ and D_5_ receptors) or D_2_-like receptors (D_2–4_ receptors) [16]. SKF83959 is a D_1_-like receptor partial agonist that has shown very high D_1_ and D_5_ receptor affinity (K_i_ = 1.18 and 7.56 nM) compared to D_2_ (K_i_ = 920 nM) and D_3_ (K_i_ = 399 nM) [35]. LE300 is a potent D_1_ and D_5_ receptor antagonist with a K_i_ of 1.9 and 7.5 nM, respectively, and demonstrated 20-fold selectivity for human D_1_-like receptors compared to D_2_-like receptors [36,37]. LE300 (150–5000 nM) also suppressed SKF38393-induced cAMP accumulation in HEK293 cells expressing D_1_ receptors [36]. In this study, higher concentrations of SKF38393 (1 µM) and LE300 (10 µM and 100 µM) were used than those used in previous studies. These concentrations were sufficient to activate or antagonize both the D_1_-like and/or D_2_-like receptors. We showed D_1_ receptor immunoreactivity in human odontoblasts in the present study. Not only D_5_ receptor, but also D_2_-like receptors, might also be expressed in odontoblasts; however, further study will be needed. It has been reported that, in line with the present results, rat odontogenic stem cells express D_1_ and D_3_ receptors. These receptors appear to be functionally involved in tooth repair by transducing DA signals from pulp-injury-activated platelets [38]. Odontoblasts in both rat incisors and molars reportedly express DA and TH, and the DA promotes the odontoblastic differentiation of pre-odontoblasts through intracellular cAMP/protein kinase A (PKA) signaling via D_1_-like receptor activation on the odontoblast [16]. In previous studies, we have shown that Ca^2+^ signals following the mechanical stimulation of odontoblasts elicit the release of ATP via pannexin-1 and glutamate via glutamate-permeable anion channels, extracellularly [6,9,39]. The released ATP, glutamate, and ADP (which is subsequently hydrolyzed from ATP) establish a paracrine signaling network between odontoblasts through the activation of ionotropic ATP (P2X) receptors, metabotropic glutamate receptors, and metabotropic ADP (P2Y) receptors, respectively; an intercellular odontoblast paracrine/autocrine communication is thereby established. The expression of both DA and TH as well as the DA receptor in odontoblasts [16], taken together with the present results, suggests that DA-induced cAMP signaling following G_s_ protein-coupled DA receptor activation may establish intercellular odontoblast paracrine and/or autocrine communication to promote odontoblastic differentiation and dentinogenesis. However, further studies are required to confirm this hypothesis.

Four adenosine receptors (A_1_, A_2A_, A_2B_, and A_3_) have been cloned in a variety of species [40]. All adenosine receptors are seven-transmembrane GPCRs linked to a variety of intercellular, intracellular, and transmembrane signal transduction pathways [40]. PSB0777 exhibited high affinity for human A_1_ (Ki = 541 nM) and A_2A_ (Ki = 360 nM) receptors, and no or negligible affinity for A_2B_ and A_3_ receptors, indicating high selectivity for human A_1_ and A_2A_ receptors [41]. In addition, PSB0777 dose-dependently accumulated cAMP with an EC_50_ of 117 nM in CHO cells stably expressing human A_2A_ receptors. The pEC_50_ for cAMP production in HEK 293T cells expressing wild-type A_2A_ receptors was 8.1 [42]. ZM241385 had a high affinity for A_2A_ receptors in guinea pigs, with a pA_2_ of 8.5. ZM241385 had a low affinity for A_1_ and A_2B_ receptors in guinea pigs with a pA_2_ of 7.06 and 5.95, respectively. ZM241385 has also shown a very low affinity for cloned rat A_3_ receptors in CHO cells, with a pIC_50_ of 3.82. Moreover, 250 nM ZM241385 reduced basolateral amygdala pyramidal cell intrinsic excitability, which is mediated by A_2A_ receptor activation [43]. In addition, 100 nM ZM241385 significantly prevented CA1 pyramidal neuronal damage caused by oxygen glucose deprivation in a model of cerebral ischemia [44]. Thus, 100 nM PSB0777 as well as 50 and 500 nM ZM241385, which were used in the present study, were sufficient to activate or antagonize A_2A_ receptors, respectively. Odontoblasts are mechanosensory receptor cells that can detect mechanical stimulation through dentinal fluid movement caused by stimuli applied to the dentin surface, and this process occurs via mechanosensitive TRP (TRPV1, TRPV2, TRPV4, and TRPA1) and Piezo1 channel activations. Ca^2+^ entry via TRP/Piezo1 channels activates the release of ATP from pannexin-1 channels into the extracellular space. The released ATP then activates the P2X receptor subtype 3 on the neurons that innervate the dental pulp, establishing intercellular synaptic-like communication between odontoblasts and neurons. This communication mediates the sensory signal transduction pathway in the generation of dentinal sensitivities, known as the ‘odontoblast mechanosensory/hydrodynamic receptor model’ [6,7,10]. The released ATP is also hydrolyzed to ADP by nucleoside triphosphate diphosphohydrolase-2 in Schwann cells or the odontoblast membrane [45]. ADP subsequently activates P2Y receptors in trigeminal ganglion (TG) neurons that innervate the dental pulp and neighboring odontoblasts to form paracrine signals. The activation of P2Y receptors establishes not only odontoblast–odontoblast but also odontoblast–TG neuron chemical communication, which may drive reactionary dentin formation and further sensory signal transduction [5,6,7,22]. Odontoblasts are known to express alkaline phosphatase (ALP) activity, which hydrolyzes ATP to adenosine; therefore, further studies should investigate whether ALP activity in odontoblasts contributes to the hydrolysis of ATP to adenosine, which then activates odontoblast A_2A_ receptors to establish inter-odontoblast paracrine communication.

VIP is a neuropeptide that is widely distributed in the central and peripheral nervous systems. VIP triggers biological responses through interactions with two receptor subtypes, VPAC_1_ and VPAC_2_, which are mainly coupled to the Gα_s_ protein and stimulate cellular AC activity [46]. VIP promotes cAMP production with an EC_50_ of 0.5–2 nM in COS cells (fibroblast-like cell lines derived from monkey kidney tissue) expressing chimeric VPAC_1_ receptors between humans and rats [47]. Applications of VIP (1–100 nM) caused reversible increases in [Ca^2+^]_i_ in gonadotropin-releasing hormone (GnRH) neurons, and increases in [Ca^2+^]_i_ did not show dose dependency [48]. VIP(6-28), has been used as a VPAC_1_ and VPAC_2_ receptor antagonist in various studies [48,49,50]. Moreover, 100 nM VIP(6-28) inhibits VIP-induced increases in GnRH neuronal firing [48]. In the present study, VIP (1 nM) and VIP(6-28) (10 nM and 100 nM) were used, and the concentrations of these reagents were appropriate for activating or inhibiting the VIP receptors. Neuropeptide VIP immunoreactivity is present in human dental pulp [51] and in TG neurons. VIP-positive nerve fibers project into the odontoblastic region and in the vicinity of blood vessels. Additionally, the levels of VIP in dental pulp tissue are higher in moderately carious teeth than in non-carious and grossly carious teeth [52]. Although further studies are needed to confirm this hypothesis, the distribution of VIP in dental pulp tissue of carious teeth and/or VIP released from nerve endings near the odontoblastic region may affect odontoblasts via the VIP–VPAC_1_ receptor axis, enhancing tertiary dentin formation and/or modulating the tooth pain threshold. We have previously shown that the mechanical stimulation of peptidergic C neurons of TG, which mimics increasing tissue pressure due to dental pulp inflammation, elicits CGRP release [13]. The released CGRP increases intracellular cAMP levels in rat odontoblasts via the CGRP–CGRP receptor axis, which may establish axon reflex in dental pulp. Additionally, TG neurons are capable of releasing adrenomedullin to establish intercellular neuron–endothelial cell communication as an axon reflex (our personal communication from YS). Therefore, our ongoing research will focus on the possible modification of odontoblast and/or dental pulp function(s) mediated by intercellular ‘retrograde’ communication from neurons to the odontoblasts via the VIP–VPAC_1_ receptor axis.

Serotonin (5-hydroxytryptamine; 5-HT) is a tryptophan-derived bioamine that acts as a neurotransmitter and mediates a wide variety of behavioral and physiological processes, such as sleep, memory, cognition, appetite, and mood. Peripheral 5-HT stored in platelet-dense granules is involved in the regulation of cardiovascular, gastrointestinal, smooth muscle, and endocrine functions [53]. 5-HT receptors are classified into seven main types (some are further divided into subtypes): 5-HT_1_ (5-HT_1A/1B/1D/1F_), 5-HT_2_ (5-HT_2A/2B/2C_), 5-HT_3_ (5-HT_3A/3B/3C_), 5-HT_4_, 5-HT_5_ (5-HT_5A/5B_, with 5-HT_5A_ being functional but 5-HT_5B_ being lost during mammalian evolution), 5-HT_6_, and 5-HT_7_ [54,55]. BIMU8 has shown a high affinity for guinea pig 5-HT_4_ receptors (pK_i_ = 7.6–7.9; where K_i_ indicates dissociation constant in the radiographic binding assay) [56]. BIMU8 dose-dependently promoted 5-HT_4_ palmitoylation with an EC_50_ of 10 nM in *Spodoptera frugiperda* cells expressing 5-HT_4_ receptors [57]. It has been demonstrated that GR113808 is a highly potent and selective 5-HT_4_ receptor antagonist with pA_2_ being 9.2 in the guinea pig proximal colon and 9.5 in the rat esophagus, respectively (A_2_ indicates dissociation constant for antagonist) [56]. 5-HT (50 µM)-induced inward currents were inhibited by 5–10 µM GR113808 in mouse CA1 hippocampal neurons and rat hypothalamic paraventricular nucleus magnocellular neurons [58,59]. In the present study, BIMU8 (10 nM) and GR113808 (5 µM and 50 µM) were used as a 5-HT_4_ receptor agonist and antagonist, respectively. Each concentration of these reagents was adequate to activate or antagonize 5-HT_4_ receptors. Recently, activated blood platelets have been reported to release 5-HT and DA during dental pulp injury [60]. The co-released 5-HT and DA are necessary for recruiting odontogenic stem cells for tooth repair through signaling via 5-HT receptor subtypes (5-HT_1D_, 5-HT_2B_, and 5-HT_7_) and DA receptor subtypes (D_1_ and D_3_). Therefore, the 5-HT and DA released from platelets during pulp injury may possibly also activate G_s_ protein-coupled 5-HT_4_ receptors (which we examined in this study) in odontoblasts to regulate cellular function.

Overall, further studies need to clarify the cellular function(s) mediated by intracellular cAMP signaling following G_s_ protein-coupled receptor activation in odontoblasts, as well as to explore the origin of ligand synthesis in dental pulp, such as endothelial cells, fibroblasts, nerve terminals, or blood cells, for odontoblasts expressing the GPCR-regulated AC signal transduction pathway. Clarification of the intercellular communication pathways between ligand-releasing cells and odontoblasts is also of immense interest.

In conclusion, here, functional IP, 5-HT_4_, D_1_, A_2A_, and VIP receptor expression in human odontoblasts was demonstrated, which activates the intracellular cAMP signaling pathway.

## Data Availability

All data are presented in the article.
